# A GPU-based algorithm for fast node label learning in large and unbalanced biomolecular networks

**DOI:** 10.1186/s12859-018-2301-4

**Published:** 2018-10-15

**Authors:** Marco Frasca, Giuliano Grossi, Jessica Gliozzo, Marco Mesiti, Marco Notaro, Paolo Perlasca, Alessandro Petrini, Giorgio Valentini

**Affiliations:** 1 AnacletoLab - Department of Computer Science, Università degli Studi di Milano, Via Comelico 39, Milano, 20135 Italy; 2 Department of Dermatology, Fondazione IRCCS Ca’ Granda,, Ospedale Maggiore Policlinico, Milan, 20122 Italy

**Keywords:** GPU-based Hopfield nets, Large-sized networks, Protein function prediction, Biological networks, Node label prediction

## Abstract

**Background:**

Several problems in network biology and medicine can be cast into a framework where entities are represented through partially labeled networks, and the aim is inferring the labels (usually binary) of the unlabeled part. Connections represent functional or genetic similarity between entities, while the labellings often are highly unbalanced, that is one class is largely under-represented: for instance in the automated protein function prediction (AFP) for most Gene Ontology terms only few proteins are annotated, or in the disease-gene prioritization problem only few genes are actually known to be involved in the etiology of a given disease. Imbalance-aware approaches to accurately predict node labels in biological networks are thereby required. Furthermore, such methods must be scalable, since input data can be large-sized as, for instance, in the context of multi-species protein networks.

**Results:**

We propose a novel semi-supervised parallel enhancement of COSNet, an imbalance-aware algorithm build on Hopfield neural model recently suggested to solve the AFP problem. By adopting an efficient representation of the graph and assuming a sparse network topology, we empirically show that it can be efficiently applied to networks with millions of nodes. The key strategy to speed up the computations is to partition nodes into independent sets so as to process each set in parallel by exploiting the power of GPU accelerators. This parallel technique ensures the convergence to asymptotically stable attractors, while preserving the asynchronous dynamics of the original model. Detailed experiments on real data and artificial big instances of the problem highlight scalability and efficiency of the proposed method.

**Conclusions:**

By parallelizing COSNet we achieved on average a speed-up of 180x in solving the AFP problem in the *S. cerevisiae*, *Mus musculus* and *Homo sapiens* organisms, while lowering memory requirements. In addition, to show the potential applicability of the method to huge biomolecular networks, we predicted node labels in artificially generated sparse networks involving hundreds of thousands to millions of nodes.

## Background

**Fig. 1:**
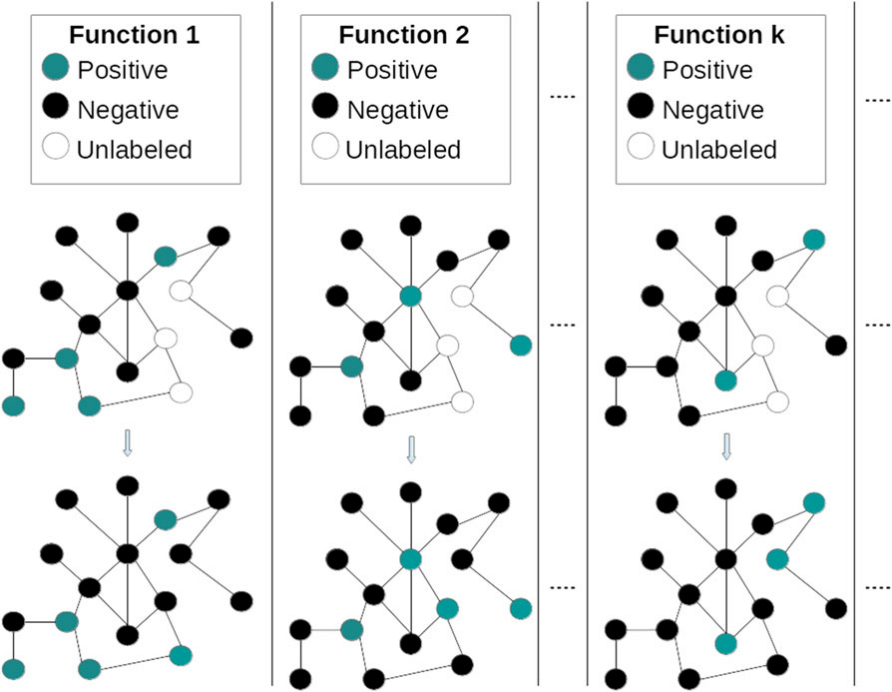
AFP problem as a set of binary classification problems. The aim is determining the color/label of unlabeled nodes/proteins, given the graph topology and the labels of the known part of the graph

The Automated Function Prediction of proteins (AFP) conveys the need of annotating the huge amount of protein sequences with their biomolecular functions. High-throughput sequencing technologies are rapidly increasing the gap between known protein sequences and proteins with experimentally annotated functions; indeed, more than 60 millions of protein sequences are available at the UniProt repository [1], and for instance less than 1% of these sequences have manually curated annotations in SwissProt [2]. Accordingly, the computational assignment of the biological functions to the proteins of an organism can greatly help in reducing this gap [3]. From this point of view, AFP can be modeled as a set of binary classification problems on graphs (one for every function), where nodes represent proteins, edges encode their functional similarity, and nodes are labeled according to the current function (see Fig. 1). Two main characteristics of AFP are the large imbalance between positive (proteins annotated with the function under study) and negative nodes (the remaining proteins), and the large size of the input graph, since networks can contain millions of proteins (see for instance multi-species protein networks [4]).

Numerous graph/network-based approaches have been proposed by the scientific community to deal with the AFP problem, ranging from methods relying on the guilt-by-association principle [5], assuming proteins topologically close in the graph are likely to share their functions, to label propagation algorithms based on Markov [6] and Gaussian Random Fields [7–10]. Other studies based their evaluation on global [11] and local convergence properties, the latter one exploiting Hopfield networks (HNs) [12], and parametric variants of this model [13–15], including an extension of the Hopfield model to a multi-category context [16, 17], where nodes are inherently partitionable into separated categories (e.g. in the multi-species protein networks). In addition, since most AFP approaches exploit a protein neighborhood to infer the functions of that protein, some works introduced a generalization of the notion of pairwise-similarity among nodes by taking into account the contribution of neighbors shared by nodes [18, 19]. Finally, other relevant studies adopted techniques based on random walks [20–22], kernel matrices [23, 24], communities [25] and co-citations [26].

Despite their large diversity and their effectiveness in solving the AFP problem, most of the above mentioned methods neglect the class-imbalance problem, leading to classifiers tending to learn mainly the negative class, thus often obtaining a sensible deterioration of their performance [27]. Moreover, they do not suitably scale with the input size, in terms of both memory usage and execution time, since they usually adopt matrix representations to embed the graph (without exploiting the graph sparsity), and basically utilize sequential programming in their model design. Indeed, recent and interesting works proposed to use secondary memory-based technologies to exploit the large disks available in standard computers to apply to big data standard graph-based semi-supervised learning algorithms; nevertheless, this can be done at the expense of the efficiency, since the swapping between secondary and primary memories data increases the computational burden [28].

In this study we propose PARCOSNET (Parallel COSNET), a methodology for solving AFP problem specifically designed to cope with the label imbalance problem and the big size of input data. It extends COSNET (Cost-Sensitive Neural Network) [14], a state-of-the-art semi-supervised method for AFP based on HNs. COSNET introduces a parametric HN to effectively handle the label imbalance, but its available implementation [29] still adopts a matrix representation of input data, allowing its application (on ordinary off-the-shelf computer) only to networks with few tens of thousands of nodes.

As first contribution, PARCOSNET reduces the memory requirements of COSNET by adopting a sparse representation of both network connections and node labeling, leveraging the sparsity of input graphs and the scarcity of positive proteins characterizing data in the AFP context. On the other side, the overall execution time is remarkably reduced 1) by splitting the HN dynamics over independent sets of neurons, where nodes in the same independent set are updated in parallel, and 2) by exploiting Graphics Processing Unit (GPUs) devices under the CUDA (Compute Unified Device Architecture) parallel programming model [30] to use one or multiple GPUs in parallel along with the CPU. Specifically, multiple GPU cores are assigned to a single independent set, thus updating in parallel the neurons within each independent set, while independent sets are in turn sequentially updated to preserve the HN convergence properties. Considering that usually multiple GO terms should be predicted for each protein, the proposed implementation adds another level of parallelism through the multithreading execution, where each CPU thread is given an instance of the AFP problem (a function to be predicted), and multiple threads are run in parallel (each using multiple cores in parallel). This thereby results in a noticeable speed-up with regard to the original COSNET implementation.

We evaluated the PARCOSNET gain in terms of both memory requirements reduction and execution speed-up by testing COSNET and PARCOSNET in predicting the Gene Ontology functional terms [31] for three eukaryotic organisms. Furthermore, synthetic graphs of different sizes, from hundreds of thousands to millions of nodes, and of different densities have been generated to empirically show the applicability of PARCOSNET on large networks.

PARCOSNET source code has been publicly released for evaluation and testing purposes, and is available on the official AnacletoLab GitHub repository, at [32].

## Methods

### Experimental data

This section is devoted to the description of both real and artificial networks used in throughout the paper.

**Real data.** Three organisms have been considered for the AFP problem, namely *Homo sapiens* (human) and two model organisms *S.cerevisiae* (yeast) and *Mus musculus* (mouse). The input networks have been retrieved from the STRING database, version 10.0 [4]: the STRING networks are highly informative networks merging several sources of information about proteins, coming from databases collecting experimental data like BIND, DIP, GRID, HPRD, IntAct, MINT or from databases collecting curated data such as Biocarta, BioCyc, KEGG, Reactome. The total number of proteins is 6391, 21151 and 19576 for yeast, mouse and human organisms, respectively.

All networks have one large connected component, with human and mouse networks with one or more smaller connected components. Furthermore, the human network is the most compact, having the smallest ratio between the number of nodes and the network diameter (see Table 1 for the network topological characteristics).

**Table 1:** Characteristics of protein networks

Organism	Nodes	Average degree	Components	Largest component size	Diameter	Weighted diameter
Yeast	6391	314.0563	1	6391	6	1.0925
Mouse	21151	596.3804	21	21105	9	1.8362
Human	19576	579.9477	2	19574	6	1.0302

Column Components denotes the number of connected components in the network, whereas Largest component size is the number of nodes in the largest connected component. Diameter is the number of edges on the longest path between two nodes, without considering edge weights

In the STRING database each protein-protein connection is associated with a confidence score: in principle, discarding edges with confidence score lower than a fixed threshold would allow to select more reliable connections; on the other side, it would generate isolated proteins (proteins with no connections), which accordingly should be discarded from the analysis. Since the aim of this study is supplying a methodolgy able to work on large networks, no edge threshold has been applied, thus including all available proteins.

Protein networks have been normalized as follows: denoted by $\hat {W}$ the matrix obtained from the STRING connections, the final network *W* is obtained by applying the normalization

$$\begin{array}{*{20}l} W = {D}^{-1/2} \hat{W} D^{-1/2}, \end{array} $$

where *D* is a diagonal matrix with non-null elements $d_{ii} = {\sum \nolimits }_{j} \hat W_{ij}$. Note that *W* is still symmetric.

Protein functional annotations have been retrieved from the Gene Ontology (GO) database, using the UniProt GOA releases 69 (9 May 2017), 155 (6 June 2017) and 168 (9 May 2017) respectively for yeast, mouse and human organisms. The GO terms have been selected from all the three branches *Biological Process* (BP), *Molecular Function* (MF), and *Cellular Component* (CC), by considering terms with at least 50 annotated proteins with experimental evidence, in order to obtain a minimal amount of information for the prediction of GO terms. The number of the resulting GO terms is summarized in Table 2. The mapping from UniProt to STRING protein identifiers was carried out according to the mapping files provided at UniProt repository.

**Table 2:** Number of GO terms with at least 5 annotations

Organism	CC	MF	BP
Yeast	50	74	191
Mouse	85	112	733
Human	115	193	580

**Artificial data.** In order to assess the performances in terms of computational time and memory consumption over larger datasets, the parallel implementation has also been tested on several artificial datasets which have been randomly generated. For random graphs we use the Erdős model in which the graph size *n* and the probability *p* to have an edge between a pair of nodes are fixed. In particular, the range chosen for *n* goes from 5·10^5^ to 1.5·10^6^, while for *p* we chose values so as to reproduce a graph density *σ*=*np* close to that of real biological network. Edge weights were uniformly generated in [0,1], while the corresponding set of labels has been generated so as to respect the unbalancing of realistic cases.

We run PARCOSNET, with the resulting 9 artificial datasets and recorded the computation time and memory consumption.

### Automated protein function prediction

In the context of the automated protein function prediction (AFP), proteins are represented by a set of nodes *V*={1,2,…,*n*}, and relationships between proteins are encoded through a symmetric *n*×*n* real weight matrix *W*, whose elements *w*_*ij*_ represent functional similarities between pairs (*i,j*) of proteins.

For a given functional class, the nodes *V* are labeled with {+,−}, leading to the subsets *P* and *N* of positive and negative nodes. For most existing taxonomies for AFP usually the functional labeling is known only for a subset *S*⊂*V*, while is unknown for *U*=*V*∖*S*. Moreover, let be *S*^+^=*S*∩*P* and *S*^−^=*S*∩*N*.

The *Automated protein function prediction problem* consists in finding a bipartition (*U*^+^,*U*^−^) of *U*, where *U*^+^ and *U*^−^ are the subsets of unlabeled proteins considered as candidate for the classes *U*∩*P* and *U*∩*N*, respectively. From this standpoint, AFP is set as a semi-supervised learning problem on graphs, since protein functions can be predicted by exploiting both labeled and unlabeled nodes/proteins and the weighted connections between them [33].

### COSNET

COSNET (COst Sensitive neural Network) [13, 14] is a neural algorithm recently proposed to face with the AFP problem. More specifically, this technique relies on a parametric family of the Hopfield model [34], where the network parameters are learned to cope with the label imbalance and the network equilibrium point is interpreted to classify the unlabeled nodes.

Formally, for a given a set of nodes *V*={1,…,*n*}, COSNET is a triple $\mathcal {H}=\langle W, \boldsymbol {\lambda }, \rho \rangle $, where:

- $W\in \mathbb {R}^{n\times n}$ is a symmetric weight matrix whose elements *w*_*ij*_∈ [0,1] represent the connection strength between the neurons (nodes) *i* and *j* (naturally *w*_*ii*_=0),
- $\boldsymbol {\lambda }=\{\lambda _{1}, \ldots, \lambda _{n}\}\in \mathbb {R}^{n}$ denotes the neuron activation thresholds,
- $\rho \in \ [\!0,\frac {\pi }{2})$ is a parameter which determines the two neuron activation (state) values {sin*ρ*,− cos*ρ*}.

The rationale of the parameter *ρ* is to conceptually separate node labels and neuron activation values, since for classical HNs activation values are in the set {−1(0),1}, that means node labels and neuron activation values coincide. Thus, appropriately learning the parameter *ρ* allows the algorithm to counterbalance the large imbalance towards negatives (see [14]).

A relevant issue for the correct design of this kind of recurrent neural networks is the synchronization of its computing nodes. The Hopfield model is a discrete-time dynamical system which admits synchronous or asynchronous updating or even both if an hybrid setting is admitted. In case of asynchronous (sequential) updating, each unit is updated independently from the others at any time *t*. Thus, by denoting with *π*=*π*(1),⋯,*π*(*n*) an arbitrary permutation on nodes *V* and with *x*_*π*(*i*)_(*t*) the state of neuron *π*(*i*) at time *t*, the dynamics assumes the form:

1 $$ x_{\pi(i)}(t+1) = \left\{\begin{array}{ll} \sin\rho,& \text{if}\, h_{\pi(i)}(t+1)\geq 0\\ -\cos\rho,& \text{otherwise} \end{array}\right.  $$

where

$$\begin{array}{*{20}l} h_{\pi(i)}(t+1)=&\sum\limits_{j=\pi(1)}^{\pi(i-1)} w_{\pi(i)j}x_{j}(t+1) \\ &+\sum\limits_{j=\pi(i+1)}^{\pi(n)} w_{\pi(i)j}x_{j}(t)-\lambda_{\pi(i)}. \end{array} $$

The convergence properties depend on the weight matrix structure *W* and the rule by which the nodes are updated. In particular, if the matrix is symmetric, it has been proved that the network converges to a stable state when operating in asynchronous mode, while it converges to a cycle of length at most 2 when operating in a synchronous (fully-parallel) mode. The proof of these properties is grounded on the so-called energy function, which is non decreasing when the state of the network ***x***=(*x*_1_,*x*_2_,…,*x*_*n*_) changes as a result of a computation (1). Since the energy function is upper-bounded, it follows that the system will converge to some state. In the classic discrete Hopfield model the energy function has the following quadratic form:

2 $$ E(\boldsymbol{x}) = -\frac{1}{2} \boldsymbol{x}^{T} W \boldsymbol{x} + \boldsymbol{x}^{T}\boldsymbol{\lambda}.  $$

As a major result, it has been shown that (2) is a Lyapunov function for the Hopfield dynamical systems with asynchronous dynamics, i.e., for each *t*>0,*E*(***x***(*t*+1))≤*E*(***x***(*t*)) and exists a time $\bar t$ such that $E(\boldsymbol {x}(t))=E(\boldsymbol {x}(\bar t))$, for all $t\geq \bar t$. Moreover, the reached fixed point $\boldsymbol {\hat {x}}=\boldsymbol {x}(\bar t)$ is a local minimum of (2).

The overall scheme of the COSNET algorithm adopting dynamics (1) can be sketched as follows: INPUT. A symmetric weight matrix *W*∈ [ 0,1]^*n*×*n*^, a labeling function *y*:*V*→{+,−}; the subsets *S*^+^,*S*^−^ and *U* of positive, negative and unlabeled instances, respectively; an initial value ***x***(0)∈{sin*ρ*,− cos*ρ*,0}^*n*^; a permutation *π* on the set *U*. Step 1. Learn the parameters $\rho =\bar \rho $ and $\lambda _{i} = \bar \lambda \in \mathbb {R}$ on the sub-network restricted to labeled nodes *S* such that the state represented by known labels is “as close as possible” to a minimum of the network energy. Step 2. Regularize the network dynamics in order to prevent the network stucking into trivial energy minima by suitably changing the thresholds and the connection weights (see [14] for more details). Hereafter, abusing notation, we assume this regularization is embedded in the connections *w*_*ij*_ and thresholds *λ*_*i*_, fer each *i,j*∈*V*. Step 3. Run the sub-network restricted to unlabeled nodes embedding the learned parameter $\bar {\rho }$ and $\bar {\lambda }$ until an equilibrium state $\hat {\boldsymbol {u}}$ is reached. On the base of state $\hat {\boldsymbol {u}}$ compute the bipartition (*U*^+^,*U*^−^) of *U* into the positive and negative neurons. OUTPUT. A bipartition (*U*^+^,*U*^−^).

Step 1 and 2 allow the method to deal with labeling imbalance, since the first step counterbalances the predominance of negatives in the node neighborhood, whereas the second step ensures to avoid trivial states composed of all negative predictions.

### Parallel COSNET

The goal of this work is to speed-up the COSNET algorithm by introducing a partial synchronous updating of the computational units in order to parallelize the network evolution stage while preserving the asynchronous dynamics. Since in AFP context the weight matrix *W* is usually sparse, several nodes are independent from each others.

The basic idea is thereby to partition the nodes belonging to the undirected graph $\mathcal {G}=\langle V,E_{W}\rangle $ (with edge set *E*_*W*_ induced by *W*) into a small number of independent subsets, which can be done recasting the problem as a vertex coloring problem of $\mathcal {G}$.

Let a vertex coloring be a map *σ*:*V*→*C*, where *C* is a set of distinct colors. We say that a coloring is proper if adjacent vertices of $\mathcal {G}$ receive distinct colors of *C*, which in turn means that if (*i,j*)∈*E*_*W*_, then *σ*(*i*)≠*σ*(*j*). Clearly, in any proper vertex coloring of $\mathcal {G}$ the vertices that receive the same color are independent. A *k*-coloring of a graph $\mathcal {G}$ is a vertex coloring of $\mathcal {G}$ that uses at most k colors and $\mathcal {G}$ is said to be *k*-colorable if it admits a proper vertex coloring using at most *k* colors. Hereafter, we denote a proper *k*-coloring by a partition $\mathcal {P}=\lbrace V_{1},\dots,V_{k}\rbrace $ of the vertex set *V* into *k* independent subsets.

Under this setting it is easy to show that by simultaneously updating the nodes of each element *V*_*i*_ of $\mathcal {P}$, one at a time, whatever the permutation of {1,…,*k*} is, the Hopfield network asynchronous dynamics is preserved. This implies that, given an initial state, the network is guaranteed to converge to a unique fixed point which is a local minimum of (2). To see that this property holds, we can start by observing that each partition $\mathcal {P}$ induces a permutation $\pi ^{\mathcal {P}}$ (that for sake of notation we simply denote by *π*) on the set *V* such that, for all *i*=1,…,*k*, the subsequence *π*_*i*_=*π*_*i*_(1),…,*π*_*i*_(*n*_*i*_) collects the nodes within *V*_*i*_={*π*_*i*_(1),…,*π*_*i*_(*n*_*i*_)}, being *n*_*i*_=|*V*_*i*_|. The permutation *π*, for a fixed $\mathcal {P}$, is then written as the juxtaposition of all subsequences *π*=*π*_1_,⋯,*π*_*k*_.

It is easy to see now that, synchronously updating all nodes in the subsequence *π*_*i*_, but asynchronously accordingly to an arbitrary permutation *ω*=*ω*(1),…,*ω*(*k*) on all subsequence indexes {1,…,*k*}, the constraints prescribed by the asynchronous updating rule are respected. Indeed, given the permutation *π*(*ω*)=*π*_*ω*(1)_,⋯,*π*_*ω*(*k*)_ induced by a *k*-coloring $\mathcal {P}$ and the permutation *ω* of the partition indexes, by applying the following partially-parallel update

3 $$ \forall u\in\pi_{\omega(i)},\quad x_{u}(t+1) = \left\{\begin{array}{ll} \sin\rho,& \text{if}\ h_{u}(t+1)\geq 0\\ -\cos\rho,& \text{otherwise} \end{array}\right.  $$

where

4 $$\begin{array}{*{20}l} h_{u}(t+1)=&\sum\limits_{v\in\pi_{\omega(1)}\cdots\pi_{\omega(i-1)}} w_{uv}x_{v}(t+1)\\ &+\sum\limits_{v\in\pi_{\omega(i+1)}\cdots\pi_{\omega(k)}}^{n} w_{uv}x_{v}(t)-\lambda_{u}. \end{array} $$

done in parallel on *u* ∈*π*_*ω*(*i*)_ and sequentially over *π*_*ω*(1)_,⋯,*π*_*ω*(*k*)_, we obtain the same attractor as in (1) with sequential update dictated exactly by *π*=*π*(*ω*).

Therefore, the following PARCOSNET algorithm - which stands for “Parallel COSNet” - encompassing a graph coloring strategy and adopting the Hopfield dynamics (3), can be sketched as follows: INPUT. Idem as in COSNET; a *k*-coloring $\mathcal {P}=\lbrace U_{1},\dots,U_{k}\rbrace $ of *U* inducing the permutation *π*; a permutation *ω* on the first *k* integers. Step 1. Idem as in COSNET. Step 2. Idem as in COSNET. Step 3. Run the sub-network restricted to unlabeled nodes embedding the learned parameters $\bar \rho, \bar \lambda $ and following updating (3) in parallel for nodes in *U*_*i*_ and sequentially across sets *U*_*i*_ by following the update permutation *π*(*ω*)=*π*_*ω*(1)_,⋯,*π*_*ω*(*k*)_ until an equilibrium state $\boldsymbol {\hat {u}}$ is reached. On the basis of state $\boldsymbol {\hat {u}}$ compute the bipartition (*U*^+^,*U*^−^) of *U* into the positive and negative neurons. OUTPUT. A bipartition (*U*^+^,*U*^−^).

Despite the additional computational cost for finding a suitable *k*-coloring, PARCOSNET shows a significant performance speed-up compared to original algorithm, mainly due to the structure of partition $\mathcal {P}$ and the number of processors $\mathcal {N}$ at hand. Although in many applications it is required to find a partition $\mathcal {P}$ having minimum cardinality (also called the chromatic number of $\mathcal {G}$), our parallelization task is instead aimed at making the evolution of the Hopfield neural system in COSNET as fast as possible; therefore, we rather seek a *k*-coloring able optimize the computational efficiency of the processors. In the following we discuss some implementation issues regarding a specific parallel architecture provided by GPUs rather than to carry out cost analysis for abstract theoretical models.

### GPU Implementation

Originally designed for graphics applications, GPUs have gradually acquired increasing importance for scientific computing and computer simulations and their processing power is one order of magnitude higher than current generation CPUs. This considerable computational power, due to specific hardware design, has paved the way for big data applications. For instance, in many domains it is frequent to address problems on very large graphs, often involving millions of vertices and graphics accelerator hardware has become a cost-effective parallel platform to solve them [35].

For this reasons, an implementation of PARCOSNET specifically targeted to NVIDIA GPUs [32] has been developed for assessing the effectiveness and performances of the algorithm. This implementation has been developed under the CUDA (Compute Unified Device Architecture) programming model [30]. Also under the same programming paradigm, a parallel implementation of a Greedy Graph Coloring (GGC) algorithm has been developed for solving the graph coloring problem, as required by the PARCOSNET algorithm.

A GPU processor is composed by an array of Streaming Multiprocessors (SM), each composed by a variable number of CUDA cores, depending on the generation of the GPU. Therefore, a single GPU processor can be seen as an array of thousands of simplified processing cores capable of scheduling and concurrently running a very large number of threads. Threads are executed according to the SIMT (Single Instruction Multiple Thread) architecture, a variation of the SIMD execution model (Single Instruction, Multiple Data) in which every thread in a block is executed independently.

Both in PARCOSNET and GGC this execution model feature has been exploited to achieve a high level of fine grained parallelism: in the GGC algorithm, each node of the graph is assigned to a thread, while in the Hopfield dynamics, an entire block of threads, ranging from 32 to 512, is assigned to a neuron for updating its state. Launching a very large number of threads has a useful consequence in the CUDA programming model, since it helps to hide latencies between the processing core and the on-board video RAM, where data are stored.

One of the major difficulty in GPU programming lies in managing its complicate memory model, since data can be stored in different address spaces, each one having its own trade-offs in terms of accessing speed and size; on top of that, CUDA kernels - i.e. programs that run on the GPU - are able to access only data residing on the on-board RAM, therefore the host system must copy the relevant part of data on the GPU before the actual computation starts, and copy back the results after the computation ends. Repeatedly copying data back and forth the GPU causes latencies that slow down the computation. In PARCOSNET a good trade-off has been achieved through several strategies. First of all, to cope with the limited amount of video ram, the graph is stored in the host RAM by means of a compressed representation, such that the entire net takes roughly (2×*n*+2×*m*) doubles, being *n* and *m* respectively the number of nodes and the number of edges of the net; this is possible by exploiting the sparsity of the net. Then, only the unlabeled portion of the graph is copied into GPU memory and processed, further reducing the transmitted data volume.

Another strategy to minimize latencies occurring in the GPU programming model is to reduce the number of synchronization points between the host system and the GPU. In an ideal heterogeneous system, the host and the hardware accelerator should work independently for maximizing concurrency, but this does not hold true if the algorithm requires several synchronization points; when such events happen, one device may be put to halt, waiting for the other to reach the synchronization point, thus slowing down the computation. This was the case of the Greedy Coloring, where each iteration on the GPU required a validation on the host system. We solved this issue by writing a coloring kernel which is rather independent from the system host, exploiting CUDA Dynamic Parallelism, where each kernel can spawn a set of sub-kernels (this is opposed to the traditional kernel launching model, where only the host system is allowed to launch computational tasks on the GPU). In this way, the entire coloring task does not require any synchronization by the host, which is therefore free to accomplish other tasks and concurrency between GPU and host is maximized.

In the following subsections, the parallelization schemes and some implementation issues about the GGC and PARCOSNET algorithms are presented. Also, CPU multithreading and memory consumption are briefly discussed.

#### Parallel Greedy Graph Coloring

As highlighted in previous section, graph coloring is a key strategy to make an efficient use of parallel (SIMT) architecture because it allows to split complex tasks into small independent subtasks that can be carried out concurrently. In our setting, each subtask can be identified by the so called Maximal Independent Set (MIS) of a graph, i.e., a maximal collection of vertices *I*∈*V* subject to the restriction that no pair of vertices in *I* are adjacent. This subgraph structure is strictly connected to coloring, because it represents a common parallel approach for graph coloring, leveraging the parallel MIS algorithm as a subroutine (see the schema in Algorithm 1). In this approach, a partition $\mathcal {P}=\lbrace V_{1},\dots,V_{k}\rbrace $ is conceived as a collection of MISs yielded by subsequent call of the Luby parallel algorithm [36] (called LUBYMIS in the pseudocode) which works in a greedy fashion. The idea is that in every round *i* it finds a subset $\bar {I}\subset V$ which is an independent set. Then it adds $\bar {I}$ to the current independent set *I*, and removes $\bar {I}$ and its neighbors $\mathcal {N}(\bar {I})$ from the current graph *V*^′^ (see pseudocode). If $\bar I\cup \mathcal {N}(\bar {I})$ is a constant fraction of |*V*^′^|, then it will only needs $\mathcal {O}(\log |V|)$ rounds to determine *I*. It will instead ensure that by removing such subset from the graph, it removes a constant fraction of the edges. The final subset *I* at round *i* is then considered as set *V*_*i*_ in the partition $\mathcal {P}$.


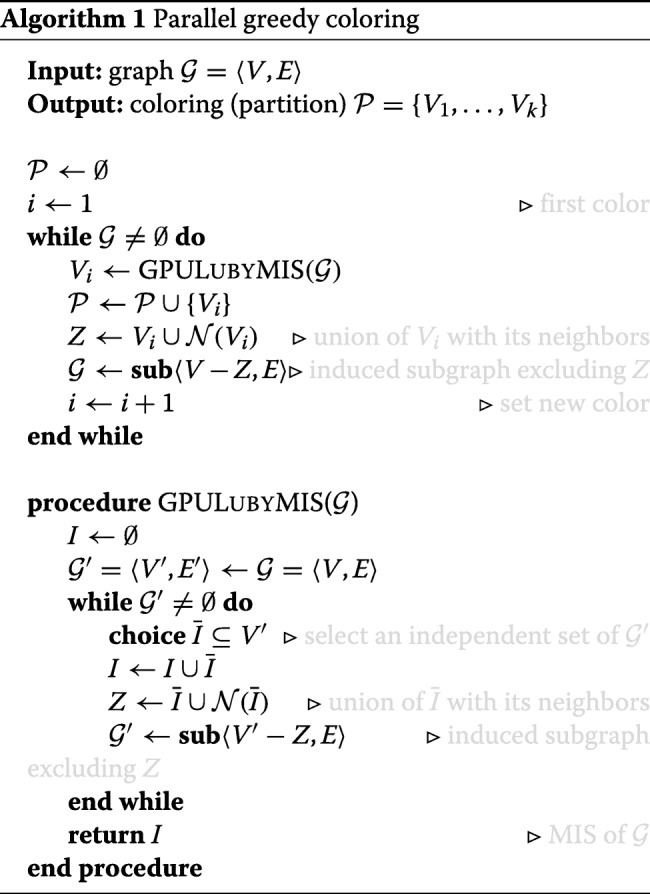


To choose $\bar {I}$ in parallel, each vertex *v* independently adds itself to $\bar {I}$ with a well chosen probability *p*(*v*). Since we want to avoid adding adjacent vertices to $\bar {I}$, we will prefer to add low degree vertices. But, if for some edge (*u,v*), both endpoints were added to $\bar {I}$, then we keep the higher degree vertex. The above strategy is concisely summed up in the statement called **choice** in the GPULUBYMIS pseudocode.

From an implementation standpoint, the latter procedure has been implemented in SIMT fashion through the curand library made available within CUDA toolkit. Each thread handles a node of the whole graph, draws a random number between 0 and 1 uniformly distributed and establishes whether it belongs or not to the MIS under construction.

#### Parallel COSNET

In this section, the implementation of the parallel Hopfield algorithm using CUDA programming model, sketched in the pseudocode of Algorithm 2, is presented and analyzed.


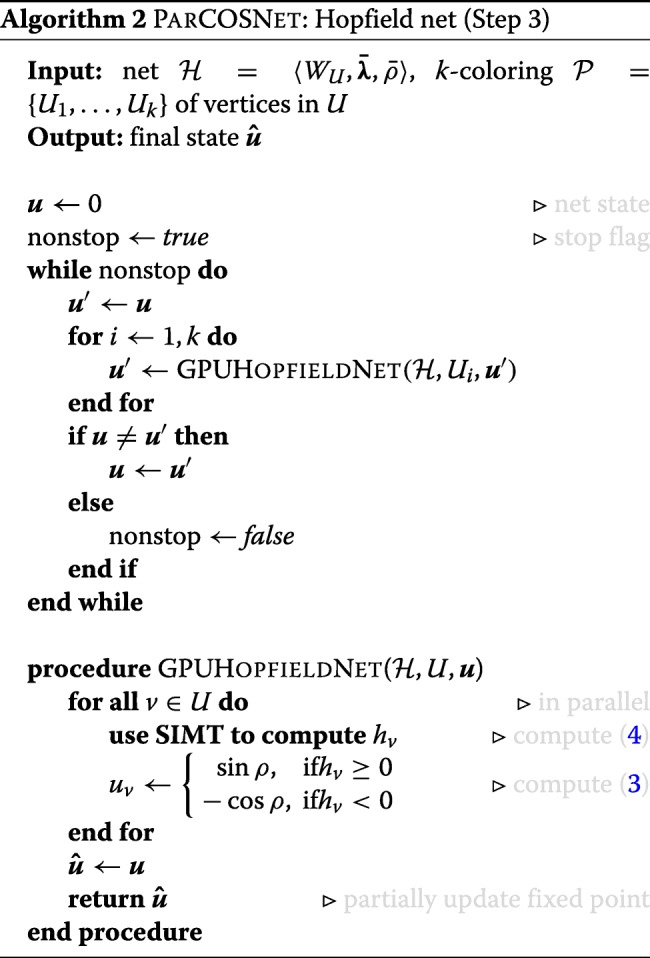


To face with the parallelization of the asynchronous dynamics (broadly described in previous sections), it should be noted that at the base of this neural architecture there is an intrinsic parallelism in the computation of the activation function (4): each single neuron implements this simple thresholding function, whose state is either “active” or “not active”. This state is determined by calculating the weighted sum of the states of its connected neurons. If the sum exceeds the threshold, the state will change to active, otherwise, the neuron will be non-active. *W*_*U*_ is the matrix of connections among nodes in *U*.

All these observations are captured in what we call GPUHOPFIELDNET procedure within the pseudocode. This algorithm naturally exposes two different levels of parallelism which can be exploited for realizing an effective scheme well suited for GPU execution. These levels correspond to two nested and different tasks: the first consists in concurrently compute the update of all nodes sharing the same color (i.e., belonging to *i*-th cluster *V*_*i*_), while the second consists in executing in parallel the addition and thresholding required for the updating of the state of each neuron. Also, the tasks are characterized by different granularity, the latter being more fine-grained than the former.

The first and more coarse-grained task, i.e. concurrently updating all the nodes having the same color, has been tackled by sequentially launching *k* different instances of the GPUHopfieldNet kernel during each iteration, being *k* the number of colors returned by the coloring algorithm. Note that this approach respects the sequential requirements of the Hopfield dynamics. Each kernel is launched with a different configuration reflecting the number of nodes belonging to each cluster *V*_*i*_. In particular, we assign |*V*_*i*_| CUDA thread blocks to the *i*-th kernel, i.e. one CUDA thread block per node.

To perform the second task, i.e. to update the state of each neuron, a fixed number *n*_*T*_ of threads (ranging from 32 to 1024) is in turn assigned to each thread block. To process the weigthed contributions of each neuron neightborhood, we proceed in SIMT fashion using shared memory among all threads in the same block and applying the known primitive called *parallel reduction*: this is a tree-based approach used within each CUDA thread block frequently applied to process very large arrays of *N* elements. It can be shown that, having at disposal *P* CUDA threads physically in parallel (*P* processors), the time complexity of the parallel reduction is $\mathcal {O}(N/P+\log N)$. By varying *n*_*T*_, the algorithm can be adapted to the density of the graph: very dense graphs will benefit from an increase of *n*_*T*_, since more threads will speed-up the evaluation of the neighborhood and the computation of the parallel reduction.

#### CPU multithreading and data representation

**Fig. 2:**
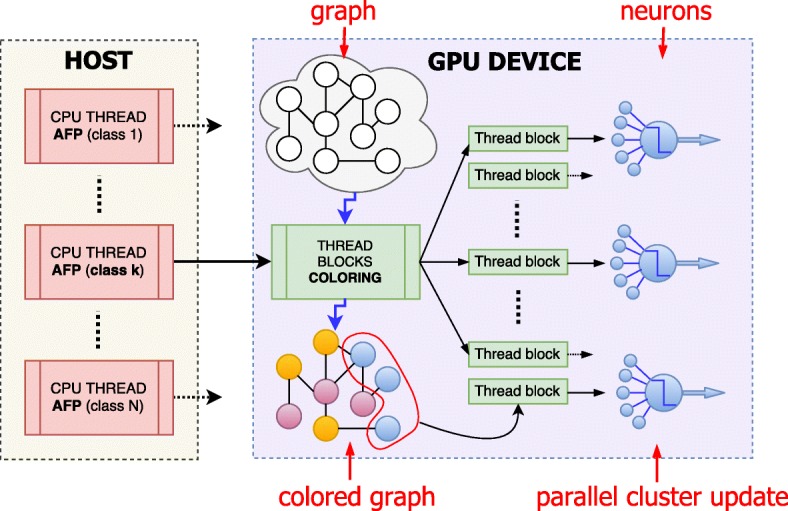
CPU/GPU schema of the PARCOSNET parallelization. Multiple CPU threads are launched in parallel each one solving the AFP problem for a given class/protein function. The GPU thread blocks, each composed of several CUDA threads, first solve the coloring problem for the graph and then concurrently process all neurons of a given color, for all colors in sequence. A further fine-grained level of parallelism is finally introduced by assigning to each neuron a thread block to perform the neuron level local computations

On top of the parallelization of the Hopfield dynamics and graph coloring, PARCOSNET exploits the independence of protein functions (target classes) to further accelerate the computation, since as stated in the introduction multiple AFP problems can be solved concurrently. As shown in Fig. 2, in PARCOSNET we put this natural additional level of parallelism by means of CPU multi-threading, where each target class is assigned to a different CPU thread. In this implementation, each thread reads a different class labeling, trains the net and then executes the coloring and Hopfield dynamics on the GPU. This dramatically improves the performances of the overall process since each AFP instance is independent, therefore multiple instances can be run concurrently. On the other side, CPU multi-threading implies to share the resources of a single GPU among multiple CPU threads, leading to serialization latencies. To deal with this problem, we exploited a CUDA compiler option that allows to assign at runtime each CPU thread to a different CUDA stream, where a CUDA stream is a queue of commands or operations that are executed in a specific order; while operations on a stream are executed sequentially, operations in different streams may be executed concurrently or out of order with respect to one another. In the specific case of PARCOSNET, kernel executions belonging to different AFP problems are interleaved and executed concurrently.

Also, the algorithm in principle could benefit of additional GPU devices to split the workload, for instance, in a system with 2 GPUs, all the odd-numbered CPU threads might offload the parallel computation to the first GPU, while the even-numbered to the second GPU.

An issue that arises when working with big datasets is memory consumption. We chose to adopt a compressed format for storing the net and the labeling *y*. Indeed, by exploiting the sparsity of *W*, for each node solely its neighbors are kept in memory at run time; moreover, leveraging the scarcity of positives, only the nodes belonging to the minority class are maintained, thus saving huge amount of memory when the input graph contains millions of nodes.

## Results

### Real data

Although COSNET has already been validated in [14, 15, 37] to solve AFP, for sake of completeness we report in Table 2 its performances in predicting the GO terms. The generalization abilities of COSNET have been assessed through a 5-fold cross validation (CV), and evaluated in terms of *Precision* (the proportion of positives correctly predicted) and *Recall* (the proportion of real positive discovered) combined in the *F* measure, which is the harmonic mean of precision and recall. In this context, where positives are rare, these measures are more informative than the error rate. Moreover, to evaluate the ability of COSNET as ranker, we also report the *Area Under the Precision Recall Curve* (AUPRC), measure adopted in the recent CAFA2 international challenge to evaluate protein ranking [38]. To provide a protein ranking for COSNET related to the current GO term, we use the neuron state at equilibrium, as done in [15, 39]. Table 3 contains the corresponding results averaged across terms in each GO branch.

**Table 3:** Average COSNET performance in predicting GO protein functions

Organism	Precision	Recall	F	AUPRC
Yeast - CC	0.3467	0.5139	0.4023	0.363
Yeast - MF	0.3317	0.4609	0.3815	0.3237
Yeast - BP	0.4042	0.5231	0.4486	0.4079
Mouse - CC	0.2068	0.2383	0.2198	0.1517
Mouse - MF	0.1861	0.2377	0.2068	0.1411
Mouse - BP	0.1473	0.1725	0.1582	0.0935
Human - CC	0.2238	0.2765	0.2448	0.1709
Human - MF	0.1847	0.2302	0.2032	0.1356
Human - BP	0.1485	0.1793	0.1611	0.0972

The COSNET implementation is publicly available as an R package [29], where the time consuming procedures (e.g. parameter learning and Hopfield dynamics) for efficiency reasons are implemented in C language. Moreover, this package adopts a matrix representation for the input network, since the R language is optimized for matrix-based computations.

Then we tested PARCOSNET on the same data sets, pointing out that its classification performances are the same as COSNET. The aim is assessing the computational speed-up achieved by PARCOSNET with respect to the already available COSNET implementation. To this end we tested both versions under the same setting, i.e. 5-fold cross validation, and the same learning parameters.

PARCOSNET has been executed using 1, 4, 8 and 12 CPU threads; to assess the scalability of the multithread approach, even the overall CPU occupancy of each execution has been computed. Also the maximum memory footprint and the execution time have been collected; the latter is used to evaluate the speed-up defined as *T*_*s*_/*T*_*p*_, where *T*_*s*_ and *T*_*p*_ are the execution times of sequential and parallel implementation, respectively.

All tests have been performed on the same host system, a workstation having 2 Intel Xeon *E*5−2620*v*3 CPUs clocked at 2.40 GHz, 64 GB of RAM memory, 2 TB disk and Linux Ubuntu 16.04 as operating system. The workstation is equipped with an NVidia GeForce *GTX*980 GPU card, featuring 2048 CUDA cores, 4 GB of dedicated on-board video memory and having Compute Capability 5.2. The C++ portion of the code has been compiled wit GCC 5.4.0, while the rest (that is, the CUDA kernels) with NVCC 8.0.44. As for COSNET, it has been executed under R version 3.4.2.

Table 4 reports the average execution time in seconds of COSNET, and PARCOSNET, for computing an entire CV cycle for a single GO term.

**Table 4:** Average CPU time in seconds for COSNET and PARCOSNET to perform a CV cycle on one GO term

Method	Yeast	Mouse	Human
COSNet	8.86	107.57	84.03
ParCOSNet	0.28	1.71	1.49
ParCOSNet 4	0.09	0.54	0.48
ParCOSNet 8	0.07	0.33	0.31
ParCOSNet 12	0.06	0.28	0.26

**Table 5:** Average speed-up *T*_*s*_/*T*_*p*_, where *T*_*s*_ and *T*_*p*_ are the average execution time of COSNET and PARCOSNET to perform an entire cross-validation on one GO term

Method	Yeast	Mouse	Human
ParCOSNet	31.64x	62.90x	56.39x
ParCOSNet 4	98.44x	199.20x	175.06x
ParCOSNet 8	126.57x	325.96x	271.06x
ParCOSNet 12	147.66x	384.18x	323.19x

To better evaluate the contribution of multithreading, Table 6 shows the average CPU occupancy of each execution of PARCOSNET. Data collected in this table is useful to assess the scalability of the parallelization over the number of CPU threads. Optimal values for this tables should be ideally near 100*%*×*n*, with *n* number of threads assigned to the task. As an example, optimal scalability for PARCOSNET executed on 12 CPU threads is achieved when occupancy reaches 1200%.

**Table 6:** Average CPU occupancy in percentage for PARCOSNET to perform a CV cycle on every GO term. Optimal scalability is achieved when the occupancy reaches 100*%*×*n*, with *n* the number of CPU threads

Method	Yeast	Mouse	Human
ParCOSNet	99%	100%	100%
ParCOSNet 4	345%	364%	368%
ParCOSNet 8	613%	659%	692%
ParCOSNet 12	815%	941%	975%

Finally, to evaluate also the memory usage, Table 7 reports the maximum memory footprint of COSNET and PARCOSNET when predicting a single GO term. For COSNET, memory usage excludes the memory required by the R interpreter itself, thus counting just the space of objects created by R and C procedures.

**Table 7:** Average memory usage in GigaBytes (GBs) for COSNET and PARCOSNET when running cross-validation to predict GO terms

Method	Yeast	Mouse	Human
COSNet	0.40	3.73	3.26
ParCOSNet	0.27	1.13	0.94

### Artificial data

Tests performed on the artificial dataset are aimed to evaluate the potential application of PARCOSNET on big data. We tested the method on single labeling, and accordingly the method has been executed in a single CPU thread mode. Table 8 shows the average execution time in seconds and the maximum memory occupancy in GB of PARCOSNET. Three dataset sizes have been considered, (each corresponding to a column in the table) and, for each size, datasets having different density (average degree per node) have been generated.

**Table 8:** Average CPU time in seconds and maximum memory consumption in GB for PARCOSNET to perform a single CV cycle on one class over the synthetic datasets

		Number of nodes		
Density (*σ*)		500k	1000k	1500k
50	Time	45.3	137	330
	Memory	1.94	3.86	5.81
100	Time	54.3	166	360
	Memory	3.69	7.37	11.1
300	Time	92.9	248	609
	Memory	10.7	21.4	32.1

## Discussion

### Real data

The time reduction obtained by PARCOSNET is impressive (Table 4): for instance on mouse data the execution time is reduced from around 107*s* to 1.71*s*, when using a single thread. Multithreading further accelerates the execution, passing to 0.54*s*,0.33*s* and 0.28*s* respectively when using 4, 8, and 12 CPU threads. To better understand these results, in Table 5 the speed-up gained by PARCOSNET when compared with COSNET is also reported. Even when using a single thread implementation, PARCOSNET achieves a speed-up of range 31.64× (yeast) and 62.90× (mouse), whereas it gains up to two order of magnitude with respect to COSNET implementation when using in multithreaded version, up to 383.18× on mouse data.

Moreover, PARCOSNET occupancy is not far from the optimal value when using 4 threads, while slightly decreasing (in proportion) when the number of threads increases (see Table 6). This is due to the fact that all the CPU threads concurrently access the same GPU, creating a minor bottleneck in computation. Indeed, we are fairly sure that adding others GPU to the host system might significantly improve the occupancy in multithread execution, since the workload can be equally divided between the devices.

PARCOSNET has been executed with 1, 4, 8 and 12 CPU threads, but only the execution of 1 thread is reported, since the allocation footprints for the 4, 8 and 12 thread execution are pretty similar: as a matter of fact, maximum memory consumption is reached before the actual computation starts, i.e. while importing and compressing the network file (Table 7). Results show that, thanks to the compressed representation of both the net and labeling, memory usage is remarkably decreased, ranging from almost the half memory used by PARCOSNET on yeast, to less than one third on human and mouse.

### Artificial data

Interestingly, PARCOSNET is able to predict node labels on graphs with 1.5 millions of nodes and average degree 300 in around 10 minutes, and using around 32 GB of RAM, nowadays available on the majority of ordinary off-the-shelf computers (Table 8). Furthermore, PARCOSNET shows a good scalability in terms of both computational time and memory consumption. The execution time increases less than linearly with the density, and little more than linearly with the number of nodes. The memory usage grows less than linearly with the number of nodes and little more than linearly with the density. This is likely due to the fact that most of the memory consumption occurs with the import and compression of the net. Nevertheless this limitation can be addressed by off-line preprocessing the data and then importing the resulting file in compressed format for PARCOSNET processing. This strategy would allow PARCOSNET to process even larger datasets: as an example, the actual memory footprint recorded during the computation of the dataset composed by 1.5 million nodes with the average density of 300 edges per nodes, is around 4 GB of RAM memory and 1 GB of GPU memory, which is a tiny fraction compared to the maximum quantity of RAM used for compressing the graph.

## Conclusions

PARCOSNET is a method for the automated function prediction (AFP), well-suited to process large protein networks with strongly unbalanced labels. PARCOSNET introduces a parallel and sparse implementation of COSNET, a state-of-art imbalance-aware method for predicting protein functions, which allows both to remarkably speeding up the computation and reducing the memory requirements. In particular, the dynamics of the Hopfield network on which COSNET builds upon is parallelized by solving a vertex coloring problem on the graph/network, partitioning nodes into sets of independent nodes which are updated in parallel by using Graphics Processing Unit (GPUs) devices and CUDA programming. By leveraging the sparsity of biological networks and of the available annotated proteins characterizing the AFP context, PARCOSNET adopts a sparse representation for both network connections and protein functions/labels. This, together with the parallel design and the usage of GPU devices, allows to significantly speed-up the computation with ordinary single-species biological networks, and opens the avenue to efficiently predict protein functions in large multi-species networks on ordinary computers, as shown in the experiments performed with synthetic networks having millions of nodes and hundreds of millions of edges.

## Abbreviations

AFP: Automated function prediction
CUDA: Compute unified device architecture
CV: Cross validation
GCC: GNU compiler collection
GO: Gene Ontology
GPU: Graphics processing unit
HN: Hopfield network
SIMT: Single instruction multiple thread
SIMD: Single instruction, multiple data
